# Molecular Epidemiology of *Neisseria meningitidis* Serogroup B in Brazil

**DOI:** 10.1371/journal.pone.0033016

**Published:** 2012-03-14

**Authors:** Ivano de Filippis, Ana Paula S. de Lemos, Jessica B. Hostetler, Kurt Wollenberg, Claudio T. Sacchi, Lee H. Harrison, Margaret C. Bash, D. Rebecca Prevots

**Affiliations:** 1 National Quality Control Institute (INCQS), Oswaldo Cruz Foundation (FIOCRUZ), Rio de Janeiro, Brazil; 2 Laboratory of Bacterial Polysaccharides, Center for Biologics Evaluation and Research (CBER), Food and Drug Administration (FDA), Bethesda, Maryland, United States of America; 3 Epidemiology Unit, Laboratory of Clinical Infectious Diseases, Division of Intramural Research, National Institute of Allergy and Infectious Diseases (NIAID), National Institutes of Health (NIH), Bethesda, Maryland, United States of America; 4 Department of Bacteriology, Instituto Adolfo Lutz (IAL), São Paolo, Brazil; 5 J. Craig Venter Institute (JCVI), Rockville, Maryland, United States of America; 6 Office of Cyberinfrastructure and Computational Biology (OCICB), National Institute of Allergy and Infectious Diseases (NIAID), National Institutes of Health (NIH), Bethesda, Maryland, United States of America; 7 Department of Immunology, Instituto Adolfo Lutz, São Paolo, Brazil; 8 Infectious Diseases Epidemiology Research Unit, University of Pittsburgh, Pittsburgh, Pennsylvania, United States of America; Health Protection Agency, United Kingdom

## Abstract

**Background:**

*Neisseria meningitidis* serogroup B has been predominant in Brazil, but no broadly effective vaccine is available to prevent endemic meningococcal disease. To understand genetic diversity among serogroup B strains in Brazil, we selected a nationally representative sample of clinical disease isolates from 2004, and a temporally representative sample for the state of São Paulo (1988–2006) for study (n = 372).

**Methods:**

We performed multi-locus sequence typing (MLST) and sequence analysis of five outer membrane protein (OMP) genes, including novel vaccine targets *fHbp* and *nadA*.

**Results:**

In 2004, strain B:4:P1.15,19 clonal complex ST-32/ET-5 (cc32) predominated throughout Brazil; regional variation in MLST sequence type (ST), *fetA*, and *porB* was significant but diversity was limited for *nadA* and *fHbp*. Between 1988 and 1996, the São Paulo isolates shifted from clonal complex ST-41/44/Lineage 3 (cc41/44) to cc32. OMP variation was associated with but not predicted by cc or ST. Overall, *fHbp* variant 1/subfamily B was present in 80% of isolates and showed little diversity. The majority of *nadA* were similar to reference allele 1.

**Conclusions:**

A predominant serogroup B lineage has circulated in Brazil for over a decade with significant regional and temporal diversity in ST, *fetA*, and *porB*, but not in *nadA* and *fHbp*.

## Introduction

*Neisseria meningitidis* causes severe invasive disease which occurs sporadically or as outbreaks and which is characterized by rapid onset, high case fatality ratio and devastating sequelae. An epidemic period of serogroup B meningococcal disease began during the 1980's with spread throughout Brazil [1]. The incidence peaked in 1996 at 7.8 cases per 100,000 persons, and the epicenter was São Paulo State, Brazil where 80% of cases were caused by serogroup B strains [2]. Our aim was to describe patterns of antigenic diversity geographically and over time in the context of a predominantly clonal epidemic.

Polysaccharide and polysaccharide-protein conjugate vaccines are effective and available for prevention of meningococcal serogroups A, C, W-135, and Y, but not for serogroup B because that capsule is poorly immunogenic. Outer membrane vesicle vaccines have been used in epidemic situations [3] but vaccines to prevent genetically diverse endemic serogroup B disease are needed. Surface-exposed protein antigens are under investigation as potential vaccine candidates; the most promising may be relatively conserved novel antigen targets identified through the use of genomic and proteomic methods [4]–[6].

Serologic and molecular epidemiologic features of serogroup B meningococcal disease have been described in individual regions of Brazil [7]–[9], but nationally representative data have not been analyzed since 1997–1998 [10]. Our goal was to determine the genetic diversity of outer membrane proteins (OMPs) among a representative sample of invasive serogroup B clinical isolates from all major geographic regions of Brazil in 2004, and among additional isolates from São Paulo from the years 1988, 1996, and 2006, which span the initiation, peak and decline of the most recent epidemic at its epicenter [2]. To accomplish this, we analyzed the sequence diversity of OMP genes *porA*, *porB*, *fetA*, and the more recently identified genes *fHbp* and *nadA* encoding human factor H binding protein (FHbp) and the invasin NadA [11]–[13]. We performed multilocus sequence typing (MLST) [14] and examined associations between inferred OMP antigen type, strain lineage, geographic region, and year.

## Materials and Methods

### Study population and selection of *N. meningitidis* isolates

In Brazil, isolates from patients with confirmed meningococcal disease are reported through the Brazilian national meningitis surveillance system. Clinical (date of onset, outcome) and demographic (age, sex, region) information on suspected patients with meningitis is routinely collected as part of this surveillance system [2].

In 2004, 3,654 confirmed cases of meningococcal disease were reported to the Brazilian Ministry of Health. Overall, 54.5% of cases were reported from the Southeast, with the remainder reported from the other regions as follows: Northeast, 19.8%; South, 15.8%; North, 6.8%; Center-West, 3.1%. Serogroup was identified for 33% (1,222/3,654), of which 52% (n = 639) were serogroup B and were considered for this study. Demographic and clinical information for patients with isolates and serogroup information was similar to those without serogroup information.

The anonymized samples analyzed in this study were a subset of all Brazilian group B *N. meningitidis* isolates received and stored by the National Reference Laboratory, Instituto Adolfo Lutz (IAL), for the year 2004. To ensure both a sufficient sample size and proportional representation within regions, only states with 10 or more serogroup B isolates during 2004 were included (Center-West region excluded), and 50% of isolates from each of these states were selected through a convenience sample, using stored samples with sufficient quantity and quality for analysis. The regional representation of selected samples was as follows: South, n = 47 (20%); Southeast, n = 92 (39.1%); North, n = 21 (9.0%); Northeast, n = 75 (31.9%).

For the temporal analysis of meningococcal disease in São Paulo, a convenience sample of isolates from three additional years representing different phases of the epidemic period were selected: 50 isolates from 1988, 50 from 1996 and 47 from 2006. Seventy-one isolates from the 2004 sample were from the São Paulo region and were also included in the temporal analysis.

### PCR amplification, primer design and sequencing strategy

Heat killed cells were sent from IAL to CBER FDA where purified genomic DNA was extracted using Qiagen® DNeasy Blood & Tissue Kit. Primers for PCR were modified from those previously described for MLST [14], *porA* [15], *porB* [16] and *fetA* [17] and were developed for *fhbp* variant 1 and *nadA* (Appendix 1). PCR primers were 5′-tagged with M13 forward and reverse oligonucleotides which were used as anchors for subsequent high throughput sequencing. Internal untagged sequencing primers were used for *porA, porB, fetA, and nadA* (Table S1). Each locus was amplified with 0.5 uM of each primer using Qiagen® HotStar HiFidelity (Qiagen, Valencia, CA, USA). Sequencing was conducted at the J. Craig Venter Institute (JCVI) on an ABI® 3770 automatic sequencer using ABI® BigDye terminator V. 3.2. in duplicate for each primer, resulting in an average of 4× coverage for MLST genes and *fHbp* and an average of 8× coverage for the remaining OMP genes. Amplification and sequencing was repeated for incomplete amplification and/or sequencing failures including amplification with non-tagged primers where needed.

### Sequence assembly and editing

Sequences were assembled with the TIGR assembler [18] and examined for at least double coverage, edited, and curated using Cloe (a multiple sequence editor heavily integrated with the TIGR database). Manual editing was used to resolve ambiguous bases, evaluate quality of 1× areas, extend or join contigs, and confirm novel sequence types (STs), rare SNPs and frameshifts. Alignments were generated using CLUSTALW [19] and locally developed Perl and shell scripts, manually curated in Bioedit [20], and trimmed in-frame internal to the primer sequences. Each contig had no ambiguous bases, 3× or greater average sequence coverage (direction independent) and/or manual inspection and confirmation of high quality 1× regions. Four thousand one hundred and forty-six or 90% of the targets were submitted to GenBank with accession numbers as follows: *abcZ*: GQ169817-GQ170194; *adk*: GQ170195-GQ170569, GU245934; *aro*: GQ170570-GQ170942, GU245933; *fumC*: GQ171288-GQ171658, GU245932; *gdh*: GQ171659-GQ172036, GU245931; *pdhC*: GQ172956-GQ173322, GU245928; *pgm*: GQ173323-GQ173644, GU245927; *porA*: GQ173645-GQ173953; *porB*: GQ173954-GQ174287; *fetA*: GQ170943-GQ171287; *fHbp*: GQ172370-GQ172679, GU245930; *nadA*: GQ172680-GQ172955, GU245929.

### Clonal complex (cc), ST and genotype determination

cc and ST were determined through the publicly available Neisseria Multilocus Sequence Typing (MLST) website (http://pubmlst.org/neisseria/) maintained at the University of Oxford, UK [21] based on the MLST method described by Maiden et al [14]. Sequence-based “antigen” types were assigned from inferred protein sequences for PorA variable region (VR)1, VR2, and VR3 (loops I, IV and V); PorB loops I, IV, V, VI, and VII; FetA allele and FHbp variable segments A, B, C and D. New STs and new OMP sequences for *porA*, *porB* and *fetA* were deposited in the *Neisseria* database [21].

### Data analysis

To identify associations of demographic characteristics with antigen types, cc, and ST, we compared the proportion of variants by patient age, sex, and geographic region for 2004 samples, and by patient age, sex and year of collection for São Paulo samples using SAS (v9.2). Statistical significance was assessed using the chi-squared test, with p≤0.05.

Phylogenetic relationships among meningococcal isolates were inferred from concatenated MLST gene DNA sequences using maximum likelihood methods. Sequences were aligned using the MUSCLE program [22]. Concatenated MLST sequences were analyzed for recombination using ClonalFrame v1.2 [23]. Maximum likelihood phylogenies were calculated using Garli v0.96 [24] for sample subsets specific for 2004 and São Paulo. A similar analysis was performed for *fHbp* with 2004 and São Paolo sequences combined due to the low degree of diversity observed.

For *nadA*, phylogenetic analysis of translated amino acid was conducted using the heuristic maximum parsimony algorithm of PAUP 4b10 [25] so that information about internal gaps could be used for phylogenetic inference. For the purpose of allele classification, the multiple sequence alignment included three NadA exemplar sequences for alleles 1, 2, and 3 [26].

This study was approved by the scientific and ethical committees of the IAL, São Paulo, Brazil and exempted from NIH IRB review by the NIH Office for Human Subjects Research (OHSR).

## Results

### Study population

Overall, 374 of the 382 isolates initially obtained could be processed and had sufficient sequence data for allele assignment for at least one MLST or OMP gene. MLST cc, based on minimum of four MLST gene sequences, was assigned using the *Neisseria* database (http://pubmlst.org/neisseria/) [21] for 354 isolates (95%), of which of 343 (96.9%) belonged to one of 6 hypervirulent clonal complexes: ST-32/ET-5 (cc32), ST-41/44 (cc41/44), ST-11/ET-37 (cc11), ST-23/A3 (cc23), ST-8/A4 (cc8), ST-269 (cc269). A total of 283 (75.7%) isolates with seven complete MLST gene sequences were assigned an MLST ST. Sixty-seven (23.7%) of those had one of 62 new STs not previously reported; an additional 19 had an ST not previously reported from Brazil. For 2004 data, the proportion with missing cc, ST or OMP sequences did not vary significantly by region. With respect to year, *fetA had* significantly more missing data for 1988 and 2006 samples (16% and 13% respectively), and *porA* had a significantly higher proportion missing for 1988 compared with other years. With respect to the OMP genes, the number of samples with sequence data sufficient to assign a type through the neisseria database were: PorB (5 loops: I, IV, V, VI, VII), 302 (81%); PorA (VR1, VR2 and VR3), 302 (81%); FetA, 334 (89%); fHbp (segments A, B,C, D), 262 (70%). Evaluable sequences for NadA were obtained from 272 (73%).

The predominant European Monitoring Group for Meningococci (EMGM) types [27] among 231 isolates with data for PorA, FetA, and ST were as follows: B: P1.19,15: F5-1: ST-33 (cc32) (24%), followed by B: P1.19,15: F5-1: ST-34 (cc32) (10.8%) and B: P1.19,15: F5-1: ST-639 (cc32) (10.4%). Other variants of the same PorA, FetA, and cc32 but with different STs comprised an additional 9.1%.

### Geographic diversity of *N. meningitidis*: 2004 isolates

**Figure 1:**
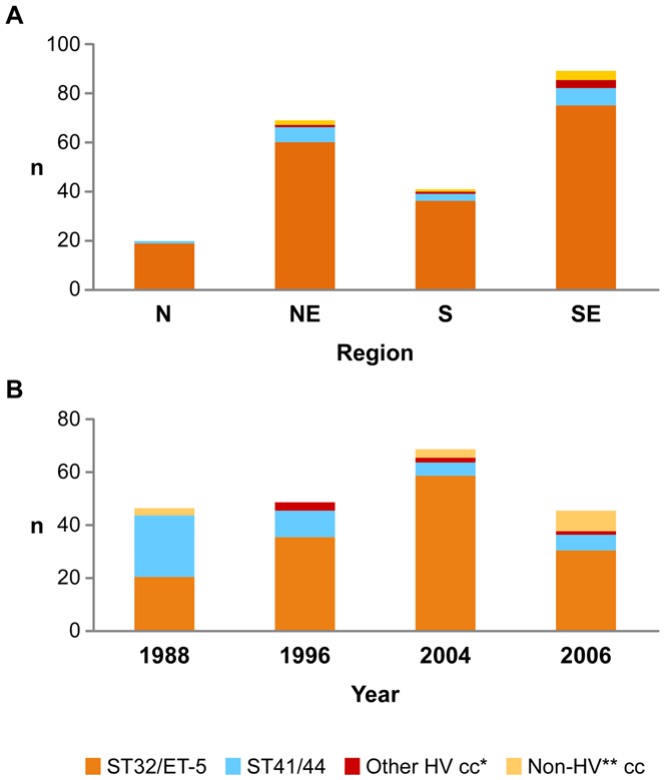
Distribution of clonal complexes by year and region in Brazil. 1a. Clonal complex by Region, Brazil 2004. 1b. Clonal complex by year, São Paulo, Brazil, 1988–2004. Footnote for Fig. 1a and 1b: *Other Hypervirulent (HV) cc = ST-8/A4, ST-23/A3; ST-11/ET-37. **Non-HV cc = ST-461,ST-103,ST-175,ST-254,ST-269,ST-35,ST-5/III; also includes STs that did not group with any known clonal complex (“unassigned”).

Patient age and sex for isolates was representative of meningitis cases in Brazil for 2004; 29% (n = 59) of isolates were from children aged ≤2 years. Overall, eight distinct cc were identified: 189 (86.3%) isolates were cc32, 17 (7.8%) were cc41/44, and the remaining 11 were cc 23 (n = 2), cc11 (n = 2), cc35 (n = 3), cc461 (n = 2), cc175 (n = 1), and cc269 (n = 1). The predominant cc in all regions was cc32 (Figure 1a). No significant differences in cc distribution by age group or region were identified.

Sixty-one STs were identified among 2004 isolates, but three STs of cc32 accounted for 55.9%: ST-33 (n = 46; 26.1%), ST-34 (n = 26; 14.7%), and ST-639 (n = 27; 15.3%). Unlike cc, the pattern of ST distribution among 2004 isolates did vary by region. In the North, ST-5996 predominated (7/13, 54%) and no isolates of ST-639 were identified. In contrast, ST-639 was common in the other regions, ranging from 10–20% of isolates. In the Northeast region, ST-33 predominated, comprising 40% of isolates while in the South and Southeast, the proportion of ST-33 (17%–20%) and ST-34 (22%-20%) were similar. Thirty-two new STs were identified among 33 strains; new STs were identified in all regions and distributed evenly across age groups.

*fetA* and *porB* also had a significantly different distribution of alleles across geographic regions. FetA 5-1 predominated in all regions except the North where 52.9% were 5–13 (Table 1). PorB loop sequence type 4,7,11,9,5 was present in 70–79% of isolates in the North and Northeast, whereas in the South and Southeast a greater number of different PorB were observed and only 55%–59% of isolates had the same predominant inferred PorB type (Table 1).

**Table 1:** PorA, PorB, FetA, FHbp by Region, 2004.

	Nn (%)	NEn (%)	Sn (%)	SEn (%)	Total
**PorA** **(VRI, VR2, VR3)**					n (%)
19, 15, 36	12 (70.6)	44 (73.3)	25 (78.1)	54 (69.2)	135 (72.2)
7-1, 1, 35-1	0	8 (13.3)	0	8 (10.3)	16 (8.6)
7, 16, 35	1 (5.8)	0	4 (12.5)	0	5 (2.7)
Other	1 (5.8)	8 (13.3)	3 (9.4)	16 (20.5)	31 (16.6)
Total	17	60	32	78	187
**PorB** * **(Loops I, IV, V, VI, VII)**					
4,7,11,9,5	15 (79.0)	42 (70.0)	20 (58.8)	42 (55.3)	119 (63.0)
4,7,31,9,5	0	7 (11.7)	0	10 (13.2)	17 (9.0)
3,7,12,11,9	0	2 (3.3)	2 (5.9)	2 (2.6)	6 (3.2)
7,7,10,12,11	0	0	6 (17.7)	1 (1.3)	7 (3.7)
9,7,13,9,12	2 (10.5)	1 (1.7)	1 (2.9)	3 (4.0)	7 (3.7)
Other	2 (10.5)	8 (13.3)	5 (14.7)	18 (23.7)	33 (17.5)
Total	19	60	34	76	189
**FetA** * **allele**					
5-1	2 (11.7)	51 (78.5)	24 (66.7)	61 (72.6)	138 (68.3)
5-13	9 (52.9)	1 (1.5)	0	1 (1.2)	11 (5.4)
3-4	2 (11.8)	0	0	0	2 (1.0)
Other	4	13	12	22	51 (25.2)
**Total**	17	65	36	84	202
**FHbp Segments** **A1-2, B1-1, C1-5, D1-5**	17 (100)	39 (84.8)	31 (86.1)	56 (91.8)	143 (86.7)
Other	0	7 (15.2)	5 (13.9)	5 (8.2)	14 (13.3)
Total	17	46	36	61	157

* p<0.05 for differences across regions.

OMP type was significantly associated with cc. Among cc32 isolates, 63% were inferred PorB type 4,7,11,9,5; 73% were PorA type 19,15,36 and 68.8% were FetA type 5-1. In contrast, within the cc41/44, only 1 (7.1%) was PorB type 4,7,11,9,5, none were PorA type 19,15,36 and none were Fet-A type 5-1. The most common 2004 type based on the European Monitoring Group for Meningococci (EMGM) classification system was B: P1.19,15: F5-1: ST-33 (cc32) (25.8%), followed by B: P1.19,15: F5-1: ST-34 (cc32) (17.7%), and B: 19,15: F:5-1: ST-639 (cc32) (9.7%), with the remainder comprising a variety of other types.

Analysis of the concatenated MLST sequences by ClonalFrame found evidence of limited recombination within individual MLST loci. Examination of the sequences found that these events were over small tracts of sequence and limited to at most one event. For these reasons recombination within MLST loci did not overwhelm the phylogenetic signal in the sequences, as demonstrated by the high bootstrap values (>70%) found in the maximum likelihood analysis.

**Figure 2:**
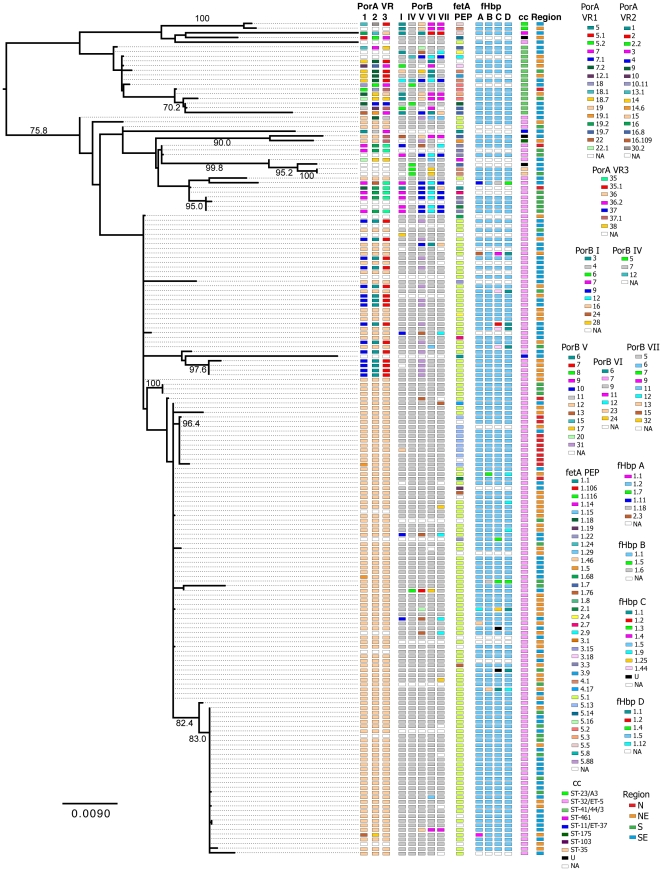
Maximum-likelihood phylogeny of the MLST DNA sequences from 2004. Annotations are for OMP types as defined by the neisseria.org (http://pubmlst.org/neisseria.org/) (PorA, PorB, FetA, and FHbp, respectively), clonal complex, and region of collection. Numbers above the branches are bootstrap percentages.

By phylogenetic analysis of concatenated MLST genes for 2004, neither the cc41/44 nor the cc32 clades were completely monophyletic. The region in which the isolates were collected did not correspond with the underlying MLST phylogenetic structure (Figure 2). While the cc32 clade had low diversity in OMP type overall, appreciable variability was observed. Notably, variability in one OMP was not predictive of diversity in other OMPs or MLST clade. Overall, the predominantly cc41/44 clade was more diverse in associated OMP types than the predominantly cc32 clade.

### Temporal diversity of *N. meningitidis:* São Paulo isolates

Among Sao Paolo isolates from 1988, 1996, 2004 and 2006, patient age (n = 175) and sex (n = 214) distributions did not vary significantly by year; and were representative of reported meningococcal disease in those years. The 111 cc32 strains were primarily ST-33 (n = 47, 42%), ST-34 (n = 12, 10.8%), and ST- 639 (n = 26, 23.4%). Both cc and ST distribution varied significantly by year of isolation: the proportion of cc32 increased from 44.7% in 1988 to 73.5% in 1996, and to 84.1% in 2004, (Figure 1b). Within cc32, the ST distribution changed significantly, particularly after 1996, with a decrease in ST-33, and an increase in other STs, particularly ST-34 and ST-639, and an increase in unique STs from 1996 to 2004 and 2006. Among the São Paulo isolates, PorA, PorB, and FetA antigen types varied significantly over time (Table 2).

**Table 2:** Por A, Por B, FetA, FHbp by Year, Sao Paolo.

	1988	1996	2004	2006	Total
**PorA** * **(VRI, VR2, VR3)**					n (%)
19,15,36	6 (22.2)	33 (70.2)	42 (70)	19 (52.8)	100 (58.8)
18-7, 9, 35-1	9 (33.3)	2 (4.3)	1 (1.7)	2 (5.5)	14 (8.2)
7-1, 1, 35-1	2 (0.62)	2 ( 4.3)	5 (8.3)	2 (5.5)	11 (6.5)
7-2, 13-1, 35-1	2 (0.62)	1 (2.1)	0	2 (5.5)	5 (2.9)
22-1, 14, 38	1 (0.37)	0	1 (1.7)	3 (8.3)	5 (2.9)
Other	16 (59.3)	9 (19.1)	11 (18.3)	8 (22,.2)	34 (20.0)
Total	27	47	60	36	170
**PorB** * **(Loops I, IV, V, VI, VII)**					
4,7, 11, 9, 5	11 (34.4)	31 (73.8)	30 (52.6)	21 (55.3)	93 (55.0)
4,7, 11, 6, 5	9 (28.1)	1 (2.4)	1 (1.8)	2 (5.3)	13 (7.7)
4,7, 31, 9, 5	1 (3.1)	1 (2.4)	6 (10.5)	3 (7.9)	11 (6.5)
4, 5, 7, 9, 5	3 (9.4)	1 (2.4)	2 (3.5)	1 (2.6)	7 (3.9)
3,7,12, 11,9	3 (9.4)	1 (2.4)	2 (3.5)	3 (7.9)	9 (5.1)
Other	5 (15.6)	7 (16.7)	16 (28.1)	8 (21.1)	36 (20.2)
Total	32	42	57	38	169
**FetA allele**					
5-1	14 (34.2)	35 (71.4)	47 (71.2)	21 (51.2)	104 (56.5)
4-1	10 (24.4)	2 (4.1)	1 (1.5)	1 (2.4)	14 (7.6)
Other	17 (41.4)	12 (24.5)	18 (27.3)	19 (46.4)	66 (35.8)
Total	41	49	66	41	184
**FHbp Segments** **A1-2, B1-1, C1-5, D1-5**	28 (87.5)	31 (96.9)	43 (93.5)	21 (75)	123 (89.1)
Other	4 (12.5)	1 (3.1)	3 (6.5)	7 (25)	15 (10.9)
Total	32	32	46	28	138

* p<0.05 for differences across years.

**Figure 3:**
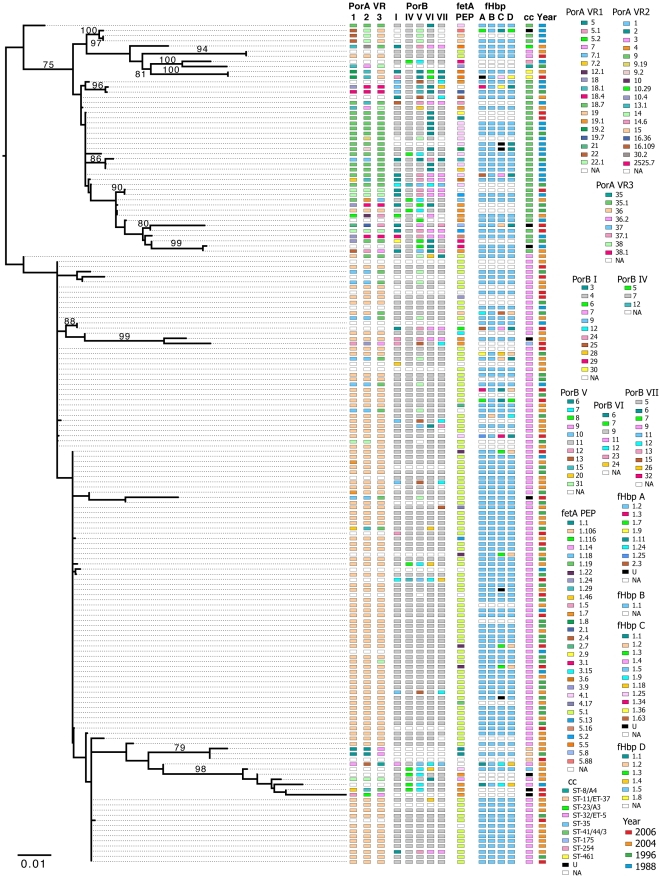
Maximum-likelihood phylogeny of the MLST DNA sequences from São Paulo. Annotations are for OMP the neisseria.org (http://pubmlst.org/neisseria.org/) (PorA, PorB, FetA, and FHbp, respectively), clonal complex, and year of collection. Numbers above the branches are bootstrap percentages.

By phylogenetic analysis of concatenated MLST genes of the São Paulo samples, the year of collection did not correspond to the branching structure of the tree (Figure 3) and the dominant STs were found in all years. As with 2004 isolates, the São Paulo OMP diversity was low overall within the cc32 clade and the diversity observed did not correspond to phylogenetic structure, year of isolation or other OMP type.

### Sequence diversity of *fHbp* and *nadA* among Brazilian group B isolates

**Figure 4:**
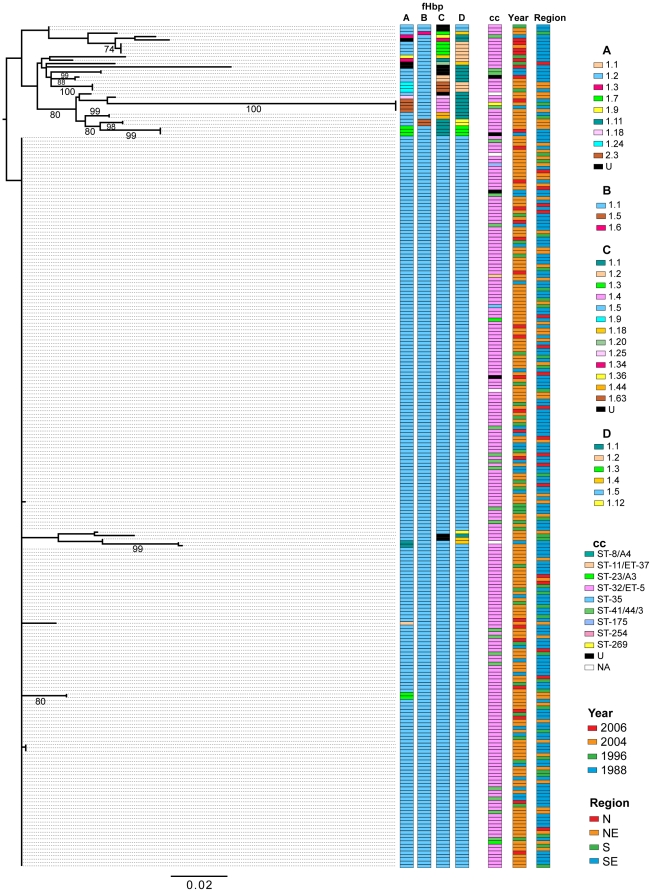
Maximum-likelihood phylogeny of the *fHbp* DNA sequences. Annotations are for FHbp segments, clonal complex, year of collection, and region of collection. Numbers above the branches are bootstrap percentages.

*fHbp* variant 1 genes were sequenced from 306 isolates (80%) and 256 (83.7%) of these were identical throughout their length to the pubMLST.org/Neisseria *fHbp* allele 311 (B24, variant 1.1, or segments A1-2, B1-1, C1.5 and D1.5.). Twenty sequences matched one of 10 other alleles available in the database, and 30 sequences had one of 20 new alleles not previously reported. A maximum-likelihood tree for 250 sequences (sequences with leading or trailing gaps were removed from the analysis) was determined using Garli v0.96 [24] (Figure 4). One large clade corresponded to allele 311 while the other major group was more variable (19 alleles among 33 samples) and contained within it one group of 4 identical sequences that were very different from the other sequences (segments A2-3, B1-1, C1-4, and D1-1). These four identical *fHbp* sequences had three different CC assignments (cc32, cc41/44, and cc269), were collected in three different years (1988, 2004, and 2006) and from three different regions (NE, S, and SE). In contrast to concatenated MLST sequences, ClonalFrame analysis of fHbp sequences identified greater evidence of recombination. The fHbp consensus tree had very little deep structure and the relationships between clades were unresolved indicating that the phylogenetic signal was obscured by recombination. For the fHbp sequences, the region estimated to have undergone recombination was approximately 33% of the total sequence.

The large majority of Brazilian *nadA* sequences (93%, 254/272) were identical to the allele 1 reference sequence (GenBank AF452481) and an additional 4% (n = 12) were highly similar but had single nucleotide insertions that resulted in early termination (n = 9) or an in-frame insertion or subtitution (n = 3). Of the six remaining sequences, one was identical to allele 3, and five were similar to either reference allele 2 or 3 but were mosaic with features showing evidence of horizontal exchange.

## Discussion

In this paper we present the first nationally representative genetic study of serogroup B *N. meningitidis* in Brazil since 1998, and the first report from Brazil that examines genetic lineages in combination with an analysis of genetic diversity among 5 OMP genes: *porA, porB, fetA, fHbp, and nadA*. We also present an historic comparison of group B strains from São Paulo for four time points spanning 18 years (1988–2006).

Previous regional reports have described a predominant Brazilian cc32 strain with phenotype, B: 4,7; P1.19,15 [1], [2], [7]–[9], [28]. A large proportion of isolates in our study had corresponding OMP sequence types: PorB loop I-4 (serotype 4) and loop VI-9 or VI-24 (serotype 7) [29], PorA VR 1–19 and VR 2–15 (serosubtype PI.19,15). However, regional differences in diversity of STs and OMPs were observed. In particular, the highest proportion of non-dominant PorA and PorB sequence types were identified in isolates from the Southeast region, and isolates from the North had a strikingly different distribution of FetA and ST. The observed regional differences, with different patterns in the North, may be because the North is remote and sparsely populated relative to the other regions, with fewer introductions and less circulation of introduced strains. The North has a population density of 3.35 inhabitants/km^2^ compared with a density of 30.7 for the Northeast and 78.2 for the Southern region [30].

Previous studies have demonstrated that a limited repertoire of antigen variants persist over time and tend to be associated with particular invasive clones [31], [32]. However, because of horizontal gene transfer, the associations are not absolute, and therefore OMP genotype cannot be inferred from cc or ST. The strong associations between FetA, PorA and PorB types within cc32, both over time and across geographic regions, are consistent with these earlier studies and the significant decrease in ST-33 and increase in unique STs after 1996 shows diversification of the epidemic clone over time. Distinct differences in OMP types and an overall greater degree of OMP diversity were observed in cc41/44 isolates relative to cc32 by categorical and phylogenetic analysis for both 2004 and São Paulo.

FHbp and NadA are under investigation as novel vaccine candidates [33]–[35] making characterization of genetic diversity relevant to estimations of vaccine coverage [36]–[38]. FHbp has been found in the majority of meningococcal strains regardless of serogroup, clonal lineage or disease/carrier origin. Recent studies in the US, Europe and South Africa [38], [39], showed that 65% to 77% of strains are variant 1 *fHbp*. In our analysis of Brazilian serogroup B meningococcal disease isolates, at least 80% of isolates had variant 1/subfamily B *fHbp* genes, and the large majority were identical to allele 311 (variant 1.1 or B24). Based on the modular nomenclature of Pajon [40], all variant 1 *fHbp* were modular group I with the exception of 4 allele 15 genes belonging to modular group IV. Geographic differences in the frequencies of modular groups have been reported, for example group IV was more frequent in the UK (23% of all isolates) than in the US (<1%) or France (6%). Strains with *fHbp* belonging to modular group IV in the UK may be associated with ccST-269, a new hypervirulent clonal complex causing disease in several European countries [41]. Based on our data, *fHbp* variant 1 is predominant among Brazilian serogroup B *N. meningitidis*, but the emergence of modular group IV should be monitored.

*nadA* was also very homogeneous in the Brazilian group B isolates. Only two *nadA* sequences clustered with allele 3 and *nadA* from four isolates were similar to allele 2 having a characteristic 7AA deletion and lacking the 47AA deletion of allele 1. Five of the six *nadA* allele 2 or 3 isolates were from recent years (2004–2006) in the S and SE regions. The remaining *nadA* were all identical or similar to allele 1. Identification of *nadA* has been reported in approximately 50% of disease-associated *N. meningitidis* overall, and 100% of strains belonging to the hypervirulent lineages cc32, cc11 and cc8, but in only 16% of carriage isolates [26]. The *nadA* gene has not been previously detected in cc41/44 isolates. However, we identified *nadA* from several cc not previously described including cc41/44 (among three new STs), cc23, and isolates of five new STs belonging to non-hypervirulent lineages. NadA expression was not studied here, but evidence of premature stop codons and variation in the TAAA repeat region preceding the start codon (data not shown) were observed. Accurate assessment of protein expression will be an essential aspect of future studies undertaken to evaluate NadA or other OMPs as vaccine targets.

We found substantially lower genetic diversity of *fHbp* and *nadA* compared to other OMPs, even correcting for the predominance of the cc32 epidemic strain. The reasons for the lower diversity among these OMP remain unclear. Diversity may be functionally limited in the context of human disease, which would support the hypothesis that they will be broadly protective as vaccine targets. Alternatively, there may be less immunologic pressure to diversify for these proteins than for the major OMPs in the context of the human host environment (predominantly carriage), either because of lower expression density on the surface, or because of alternative mechanisms of escape such as down regulation. Some diversity is clearly tolerated, and from our sequence analysis, horizontal genetic exchange plays a similar role as with other neisserial OMPs. Further study of the diversity of these novel proteins and the potential coverage of new vaccines is needed.

In summary, our study provides a comprehensive molecular analysis of ST and OMP genetic diversity of Brazilian group B meningococcal isolates. In 2004, group B meningococcal disease was predominantly caused by cc32 strains; cc and OMP type were strongly associated, but OMP diversity was not uniformly predicted by cc or other OMP type among either the geographic or the temporal samples. cc41/44 showed greater ST and OMP diversity which may be the result of an older evolutionary origin. *fHbp* and *nadA* sequences were highly homogeneous within the population of disease isolates examined. Although *N. meningitidis* serogroup C disease has increased since 2006, serogroup B continues to be a significant cause of meningococcal disease in Brazil and will remain so even if widespread use of conjugate vaccines is implemented. Greater understanding of the mechanisms of genetic diversification of serogroup B *N. meningitidis* is important for successful development, introduction, and long-term use of vaccines intended to prevent serogroup B disease.

## Supporting Information

Table S1 **Primers for MLST genes (abcZ, adk, aroE, fumC, gdh, pdhC, pgm);** ***porA*** **,** ***porB*** **,** ***fetA*** **,** ***nadA*** **(PCR and sequencing) and** ***fHbp*** **(PCR).**
(DOCX)

## References

[1] Sacchi CT, Pessoa LL, Ramos SR, Milagres LC, Camargo MCC (1992). Ongoing group B Neisseria meningitidis epidemic in São Paulo, Brazil, due to increased prevalence of a single clone of the ET-5 complex.. Journal of Clinical Microbiology.

[2] de Lemos AP, Yara TY, Gorla MC, Paiva MV, de Souza AL (2007). Clonal distribution of invasive Neisseria meningitidis serogroup C strains circulating from 1976 to 2005 in greater São Paulo, Brazil.. Journal of Clinical Microbiology.

[3] Holst J, Martin D, Arnold R, Huergo CC, Oster P (2009). Properties and clinical performance of vaccines containing outer membrane vesicles from Neisseria meningitidis.. Vaccine.

[4] Pizza M, Scarlato V, Masignani V, Giuliani MM, Arica B (2000). Identification of vaccine candidates against serogroup B meningococcus by whole-genome sequencing.. Science.

[5] Fletcher LD, Bernfield L, Barniak V, Farley JE, Howell A (2004). Vaccine potential of the Neisseria meningitidis 2086 lipoprotein.. Infection and Immunity.

[6] Giuliani MM, Adu-Bobie J, Comanducci M, Arico B, Savino B (2006). A universal vaccine for serogroup B meningococcus.. Proc Natl Acad Sci.

[7] Cordeiro SM, Neves AB, Ribeiro CT, Petersen ML, Gouveia EL (2007). Hospital-based surveillance of meningococcal meningitis in Salvador, Brazil.. Transactions of the Royal Society of Tropical Medicine and Hygiene.

[8] Baethgen LF, Weidlich L, Moraes C, Klein C, Nunes LS (2008). Epidemiology of meningococcal disease in southern Brazil from 1995 to 2003, and molecular characterization of Neisseria meningitidis using multilocus sequence typing.. Tropical Medicine & International Health.

[9] de Filippis I, Vicente AC (2005). Multilocus sequence typing and repetitive element-based polymerase chain reaction analysis of Neisseria meningitides isolates in Brazil reveal the emergence of 11 new sequence types genetically related to the ST-32 and ST-41/44 complexes and high prevalence of strains related to hypervirulent lineages.. Diagn Microbiol Infect Dis.

[10] Sacchi CT, Lemos AP, Popovic T, de Moraes JC, Whitney AM (2001). Serosubtypes and PorA types of Neisseria meningitidis serogroup B isolated in Brazil during 1997–1998: overview and implications for vaccine development.. Journal of clinical microbiology.

[11] Comanducci M, Bambini S, Brunelli B, Adu-Babie J, Arico B (2002). NadA, a novel vaccine candidate of Neisseria meningitidis.. J Exp Med.

[12] Masignani V, Comanducci M, Giuliani MM, Bambini S, Adu-Babie J (2003). Vaccination against Neisseria meningitidis using three variants of the lipoprotein GNA1870.. The Journal of Experimental Medicine.

[13] Madico G, Welsch JA, Lewis LA, McNaughton A, Perlman DH (2006). The meningococcal vaccine candidate GNA1870 binds the complement regulatory protein factor H and enhances serum resistance.. J Immunol.

[14] Maiden MC, Bygraves JA, Feil E, Morelli G, Russell JE (1998). Multilocus sequence typing: a portable approach to the identification of clones within populations of pathogenic microorganisms.. Proceedings of the National Academy of Sciences of the United States of America.

[15] Suker J, Feavers IM, Achtman M, Morelli G, Wang JF (1994). The *porA* gene in serogroup A meningococci: evolutionary stability and mechanism of genetic variation.. Molecular microbiology.

[16] Urwin R, Kaczmarski EB, Guiver M, Fox AJ, Maiden MM (1998). Amplification of the meningococcal *porB* gene for non-culture serotype characterization.. Epidemiology and infection.

[17] Thompson EA, Feavers IM, Maiden MC (2003). Antigenic diversity of meningococcal enterobactin receptor FetA, a vaccine component.. Microbiology.

[18] Sutton GG, Adams MD, Kervlage AR (1995). A new tool for assembling large sequencing projects.. Genome Science and Technology.

[19] Thompson JD, Gibson TJ, Higgins DG (2002). Multiple sequence alignment using ClustalW and ClustalX.. Current protocols in bioinformatics Chapter.

[20] Hall T (1999). Bioedit: a user friendly biological sequence alignment editor and analysis program for Windows 95/98/NT.. Nucl Acids Symp Ser.

[21] Jolley KA, Maiden MC (2010). BIGSdb: Scalable analysis of bacterial genome variation at the population level.. BMC bioinformatics.

[22] Edgar RC (2004). MUSCLE: a multiple sequence alignment method with reduced time and space complexity.. BMC bioinformatics.

[23] Didelot X, Falush D (2007). Inference of bacterial microevolution using multilocus sequence data.. Genetics.

[24] Garli website.. http://garli.googlecode.com.

[25] Swofford D (2003). Phylogenetic analysis using parsimony (and other methods).

[26] Comanducci M, Bambini S, Caugant DA, Mora M, Brunelli B (2004). NadA diversity and carriage in Neisseria meningitidis.. Infection and immunity.

[27] Jolley KA, Brehoney C, Maiden MC (2007). Molecular typing of meningococci: recommendations for target choice and nomenclature.. FEMS Microbiol.

[28] Barroso DE, Carvalho DM, Casagrande ST, Rebelo MC, Soares V (2010). Microbiological epidemiological history of meningococcal disease in Rio de Janeiro, Brazil.. The Brazilian Journal of Infectious Diseases.

[29] Sacchi CT, Lemos AP, Brandt ME, Whitney AM, Melles CEA (1998). Correlation between serological and sequencing analyses of the PorB outer membrane protein in the Neisseria meningitidis serotyping system.. Clinical and diagnostic laboratory immunology.

[30] Website for the Brazilian Institute of Geography and Statistics (IBGE).. http://www.ibge.gov.br/ibgeteen/mapas/imagens/demografia_gde.gif.

[31] Russell JE, Urwin R, Gray SJ, Fox AJ, Feavers IM (2008). Molecular epidemiology of meningococcal disease in England and Wales 1975–1995, before the introduction of serogroup C conjugate vaccines.. Microbiology.

[32] Urwin R, Russell JE, Thompson EA, Holmes EC, Feavers IM (2004). Distribution of surface protein variants among hyperinvasive meningococci: implications for vaccine design.. Infection and immunity.

[33] Findlow J, Borrow R, Snape MD, Dawson T, Holland A (2010). Multicenter, open-label, randomized phase II controlled trial of an investigational recombinant Meningococcal serogroup B vaccine with and without outer membrane vesicles, administered in infancy.. Clinical Infectious Diseases.

[34] Jiang HQ, Hoiseth SK, Harris SL, McNeil LK, Zhu D (2010). Broad vaccine coverage predicted for a bivalent recombinant factor H binding protein based vaccine to prevent serogroup B meningococcal disease.. Vaccine.

[35] Keiser PB, Biggs-Cicatelli S, Moran EE, Schmeil DH, Pinto VB (2011). A phase 1 study of a meningococcal native outer membrane vesicle vaccine made from a group B strain with deleted lpxL1 and synX, over-expressed factor H binding protein, two PorAs and stabilized OpcA expression.. Vaccine.

[36] Lucidarme J, Comanducci M, Findlow J, Gray SJ, Kaczmarski EB (2010). Characterization of fHbp, nhba (gna2132), nadA, porA, and sequence type in group B meningococcal case isolates collected in England and Wales during January 2008 and potential coverage of an investigational group B meningococcal vaccine.. Clinical and Vaccine Immunology.

[37] Bambini S, Muzzi A, Olcen P, Rappuoli R, Pizza M (2009). Distribution and genetic variability of three vaccine components in a panel of strains representative of the diversity of serogroup B meningococcus.. Vaccine.

[38] Murphy E, Andrew L, Lee KL, Ditts DA, Nunuez L (2009). Sequence diversity of the factor H binding protein vaccine candidate in epidemiologically relevant strains of serogroup B Neisseria meningitidis.. The Journal of infectious diseases.

[39] Wang X, Cohn A, Comanducci M, Andrew L, Zhao X (2009). Prevalence and genetic diversity of candidate vaccine antigens among invasive Neisseria meningitidis isolates in the United States.. Vaccine.

[40] Pajon R, Beernink PT, Harrison LH, Granoff DM (2010). Frequency of factor H-binding protein modular groups and susceptibility to cross-reactive bactericidal activity in invasive meningococcal isolates.. Vaccine.

[41] Brehony C, Jolley KA, Maiden MC (2007). Multilocus sequence typing for global surveillance of meningococcal disease.. FEMS microbiology reviews.

