# Correction: Molecular Epidemiology of *Neisseria meningitidis* Serogroup B in Brazil

**DOI:** 10.1371/annotation/6318254d-3f13-4cca-8bbe-08d0ff29aa64

**Published:** 2012-07-10

**Authors:** Ivano de Filippis, Ana Paula S. de Lemos, Jessica B. Hostetler, Kurt Wollenberg, Claudio T. Sacchi, Lee H. Harrison, Margaret C. Bash, D. Rebecca Prevots

There was an error in the author byline. Author Julie C. Dunning Hotopp was inadvertently excluded. The author's affiliation is: 5 J. Craig Venter Institute (JCVI), Rockville, Maryland, United States of America, and the author's current address is: Institute of Genomic Sciences, University of Maryland School of Medicine, Baltimore, Maryland, United States of America. The author's order in the byline is sixth and the author's contributions are: Performed the experiments, analyzed the data, and contributed reagents/materials/analysis tools. 

