# Supplementary material for: Molecular Epidemiology of Neisseria meningitidis Serogroup B in Brazil
Source: PLoS One. 2012 Mar 14;7(3):e33016. doi: 10.1371/journal.pone.0033016 (PMC3303791; doi:10.1371/journal.pone.0033016)
Supplement: Table S1 — Primers for MLST genes (abcZ, adk, aroE, fumC, gdh, pdhC, pgm); porA , porB , fetA , nadA (PCR and sequencing) and fHbp (PCR). (DOCX) [file pone.0033016.s001.docx]

**Table S1: Primers for MLST genes (abcZ, adk, aroE, fumC, gdh, pdhC, pgm), *porA, porB, fetA*, *nadA* (PCR and sequencing), and *fHbp* (PCR).**

Primer name Sequence (5`- 3`) Annealing T Product size (bp)

abcZ-P1C TGTAAAACGACGGCCAGTTGTTCCGCTTCGACTGCCAAC 50°C 898

abcZ-P2C CAGGAAACAGCTATGACCTCCCCGTCGTAAAAAACAATC

adk-P1B TGTAAAACGACGGCCAGTCCAAGCCGTGTAGAATCGTAAACC 50°C 708

adk-P2B CAGGAAACAGCTATGACCTGCCCAATGCGCCCAATAC

aroE-P1B TGTAAAACGACGGCCAGTTTTGAAACAGGCGGTTGCGG 55°C 835

aroE-P2B CAGGAAACAGCTATGACCCAGCGGTAATCCAGTGCGAC

fumC-P1B TGTAAAACGACGGCCAGTTCCCCGCCGTAAAAGCCCTG 56°C 860

fumC-P2B CAGGAAACAGCTATGACCGCCCGTCAGCAAGCCCAAC

gdh-P1B TGTAAAACGACGGCCAGTCTGCCCCCGGGGTTTTCATCT 50°C 677

gdh-P2B CAGGAAACAGCTATGACCTGTTGCGCGTTATTTCAAAGAAGG

pdhC-P1B TGTAAAACGACGGCCAGTCCGGCCGTACGACGCTGAAC 50°C 818

pdhC-P2B CAGGAAACAGCTATGACCGATGTCGGAATGGGGCAAACA

pgm-P1 TGTAAAACGACGGCCAGTCTTCAAAGCCTACGACATCCG 50°C 1339

pgm-P2 CAGGAAACAGCTATGACCCGGATTGCTTTCGATGACGGC

porA-210 TGTAAAACGACGGCCAGTATGCGAAAAAAACTTACCGCCCTC 60°C 1230

porA-211 CAGGAAACAGCTATGACCAATGAAGGCAAGCCGTCAAAAACA

porA-122L (Seq.) GGCGAGATTCAAGCCGCC

porB-PB1 TGTAAAACGACGGCCAGTTAAATGCAAAGCTAAGCGGCTTG 50°C 1040

porB-PB2 CAGGAAACAGCTATGACCTTTGTTGATACCAATCTTTTCAG

fetA-S1 TGTAAAACGACGGCCAGTCGGCGCAAGCGTATTCGG 50°C 1159

fetA-S8 CAGGAAACAGCTATGACCCGCGCCCAATTCGTAACCGTG

fetA-S13 (Seq.) TACGCAGGCAATGTAAAAGGC

fetA-S15 (Seq.) TTGCAGCGCGTCATACAGGCG

NadA-f TGTAAAACGACGGCCAGTGGCAGAATTGACATCA 42°C 1150

NadA-r CAGGAAACAGCTATGACCCGTTGTAAGGTTGGA

Nad_S1 (Seq.) TGAGCATGAAACACTTTCCA

Nad_S2 (Seq.) CATGCTTGTCGACGGTATCA

fHbp-f TGTAAAACGACGGCCAGTGCCCTGATTCTGACC 41°C 767

fHbp-r CAGGAAACAGCTATGACCCCGATATGGCGTATG

^a^M13 forward and reverse tags are underlined
